# Bayesian and Machine Learning Models for Genomic Prediction of Anterior Cruciate Ligament Rupture in the Canine Model

**DOI:** 10.1534/g3.120.401244

**Published:** 2020-06-04

**Authors:** Lauren A. Baker, Mehdi Momen, Kore Chan, Nathan Bollig, Fernando Brito Lopes, Guilherme J. M. Rosa, Rory J. Todhunter, Emily E. Binversie, Susannah J. Sample, Peter Muir

**Affiliations:** * Department of Surgical Sciences, School of Veterinary Medicine,; ^†^ Department of Animal Sciences, University of Wisconsin-Madison, WI, 53706, and; ^‡^ Department of Clinical Sciences, College of Veterinary Medicine, Cornell University, Ithaca, New York, 14853

**Keywords:** ACL rupture, Dog model, Canine, Genomic prediction, Shared data resources, GenPred

## Abstract

Anterior cruciate ligament (ACL) rupture is a common, debilitating condition that leads to early-onset osteoarthritis and reduced quality of human life. ACL rupture is a complex disease with both genetic and environmental risk factors. Characterizing the genetic basis of ACL rupture would provide the ability to identify individuals that have high genetic risk and allow the opportunity for preventative management. Spontaneous ACL rupture is also common in dogs and shows a similar clinical presentation and progression. Thus, the dog has emerged as an excellent genomic model for human ACL rupture. Genome-wide association studies (GWAS) in the dog have identified a number of candidate genetic variants, but research in genomic prediction has been limited. In this analysis, we explore several Bayesian and machine learning models for genomic prediction of ACL rupture in the Labrador Retriever dog. Our work demonstrates the feasibility of predicting ACL rupture from SNPs in the Labrador Retriever model with and without consideration of non-genetic risk factors. Genomic prediction including non-genetic risk factors approached clinical relevance using multiple linear Bayesian and non-linear models. This analysis represents the first steps toward development of a predictive algorithm for ACL rupture in the Labrador Retriever model. Future work may extend this algorithm to other high-risk breeds of dog. The ability to accurately predict individual dogs at high risk for ACL rupture would identify candidates for clinical trials that would benefit both veterinary and human medicine.

Anterior cruciate ligament (ACL) rupture is a common condition with serious long-term consequences, as up to 50% of affected individuals will develop osteoarthritis (OA) within 10 years of rupture (Lohmander *et al.* 2007). This is especially troubling given that the highest incidence is in adolescent athletes (Lohmander *et al.* 2007), who will experience a significant health burden while they are still young. The impact of this reality is reflected in the lifetime burden of ACL rupture in the United States, which is $7.6 billion annually if surgical treatment is pursued, *vs.* $17.7 billion if treatment is limited to physical rehabilitation (Mather *et al.* 2013). The vast majority of ACL ruptures occur in the absence of contact injury (Smith *et al.* 2012a), and surgical reconstruction does not consistently prevent development of OA, which supports the hypothesis that ACL rupture is at least partially due to biochemical influences. Several risk factors have been identified, including genetic predisposition (Smith *et al.* 2012a; Smith *et al.* 2012b; Kaynak *et al.* 2017). Understanding the genetic basis of ACL rupture is important, as it would allow medical professionals to identify those individuals that have a higher inborn risk of rupture. Interventions could then take place to mitigate risk and potentially prevent these people from developing ACL rupture.

Spontaneous ACL rupture is also a disease of importance in veterinary medicine, as the condition is diagnosed in 20% of dogs evaluated for lameness at university hospitals (Johnson *et al.* 1994). The American public spends greater than $1 billion annually on treatments for canine ACL rupture (Wilke *et al.* 2005). ACL rupture in dogs has a similar presentation and progression to ACL rupture in humans, including development of OA in spite of surgical stabilization (Rayward *et al.* 2004). Thus, spontaneous ACL rupture in dogs has emerged as an excellent model for ACL rupture in human beings (Gregory *et al.* 2012; Proffen *et al.* 2012). ACL rupture in dogs has particular value as a genomic model, as the condition has a marked breed-predisposition, and in some breeds, prevalence is ∼100 fold greater than in human beings (Witsberger *et al.* 2008; Gianotti *et al.* 2009). Extensive linkage disequilbrium (LD) in dogs facilitates genome-wide association study (GWAS) (Karlsson and Lindblad-Toh 2008), and multiple ACL rupture GWAS in dogs have been undertaken (Baird *et al.* 2014; Hayward *et al.* 2016; Baker *et al.* 2017; Baker *et al.* 2018). However, most of this research has focused on biological interpretation of SNPs that reach genome-wide significance, and little has been done to attempt genomic prediction of canine ACL rupture.

Genomic prediction as a method focuses less on individual SNPs and assumes that all SNP markers may be linked to causal variants, even if their effects are quite small (Meuwissen *et al.* 2016). These polygenic effects act in combination to influence risk of disease (Robinson *et al.* 2014). The number of genetic variants that are believed to affect complex traits, such as ACL rupture, has increased ∼100 fold during the last 18 years with most estimates suggesting there are thousands of small effect variants distributed across the genome (Meuwissen *et al.* 2016). SNPs with measurable effects can be used on their own to estimate genetic risk or combined with measurements of non-genetic risk factors to create absolute risk models that estimate the probability that an individual will develop the disease over time (Chatterjee *et al.* 2016). Genomic prediction in dog populations to improve veterinary health has not received much attention. One study attempted genomic prediction of canine hip dysplasia in the Labrador Retriever with moderate to poor predictive accuracy (Sánchez-Molano *et al.* 2015). The ability to predict ACL rupture in dogs would be extremely valuable from a veterinary medical perspective, but also because it would enable prospective research of interventional treatments using spontaneous ACL rupture in the dog as a model for human ACL rupture. Insights gained from research in the dog model would lead to advancements in both veterinary and human medical research.

There are multiple methods for genomic prediction. Each method has advantages and disadvantages with respect to model assumptions and how well the model fits the data. With respect to prediction of complex traits, points to consider when choosing a model include the genetic architecture of the trait in terms of the potential presence of major genes, epistatic interactions, and a polygenic component. In addition, other factors to be considered include marker density and the strength of LD among them, as well as sample size (Hayes *et al.* 2010; Pérez and de Los Campos 2014). Bayesian models lend themselves well to genomic prediction, as they have the ability to incorporate prior information about expected SNP effects, for example allowing SNPs to have varying effect sizes, which makes more sense biologically than assuming all SNPs have the same effect size (Moser *et al.* 2015; Meuwissen *et al.* 2016). Classification-based machine learning methods have also gained popularity for genomic prediction of binary traits. Here, a GWAS training set is viewed as a supervised classification problem whereby individuals are partitioned into case or control groups, and each group can be described using a combination of SNP inputs that may have one of 3 discrete values corresponding to the number of minor alleles present at each SNP (Botta *et al.* 2014). As no single model has been shown to perform best across data sets and traits (Pérez and de Los Campos 2014), the following analyses were performed to investigate the feasibility of genomic prediction of ACL rupture in the dog model using several Bayesian and machine learning approaches. We provide insight on which methods appear to be most suitable for genomic prediction of a complex trait disease in purebred dogs, and potential and future directions for development of a predictive genetic test for ACL rupture.

## Materials and Methods

### Data collection and phenotyping

Client-owned Labrador Retrievers were recruited from the UW-Madison Veterinary Care teaching hospital and through online advertising. All owners gave informed consent to participate in the study. When possible, a four-generation pedigree was obtained to confirm purebred status. Each dog was carefully phenotyped through orthopedic exam (Muir 1997) and lateral stifle radiographs. ACL rupture in affected dogs was verified during surgical treatment. Dogs classified as controls were over the age of 8 years, negative for palpable knee laxity, and showed no signs of joint effusion or osteophytosis that would be consistent with ACL rupture on lateral stifle radiographs (Chuang *et al.* 2014). This age cutoff was chosen because Labrador Retrievers 8 years of age and older have approximately a 6% chance of developing ACL rupture (Reif and Probst 2003). DNA was isolated from saliva or blood samples obtained in accordance with the Guide for the Care and Use of Laboratory Animals with approval from the Institutional Animal Care and Use Committee of the School of Veterinary Medicine, University of Wisconsin-Madison. SNP genotyping was performed using the Illumina Canine HD BeadChip, which contains approximately 230,000 SNPs distributed evenly across the canine genome (CanFam3.1). The Wisconsin dataset contained 336 dogs (134 cases, 202 controls). This study also used public data from a recent study that used the same genotyping platform (Hayward *et al.* 2016) to increase sample size by 287 Labrador Retriever dogs. The final dataset consisted of genotyping data and covariates on 622 Labrador Retriever dogs (247 cases, 375 controls).

### SNP genotyping quality control

Genotype data were filtered with PLINK for quality control (Chang *et al.* 2015). All samples had a genotyping call rate >95%. SNPs were excluded if minor allele frequency (MAF) was less than or equal to 0.05, if genotyping rate was less than or equal to 95% or if there was deviation from Hardy-Weinberg proportions at *P* < 1E-07.

### Experimental design

**Figure 1:**
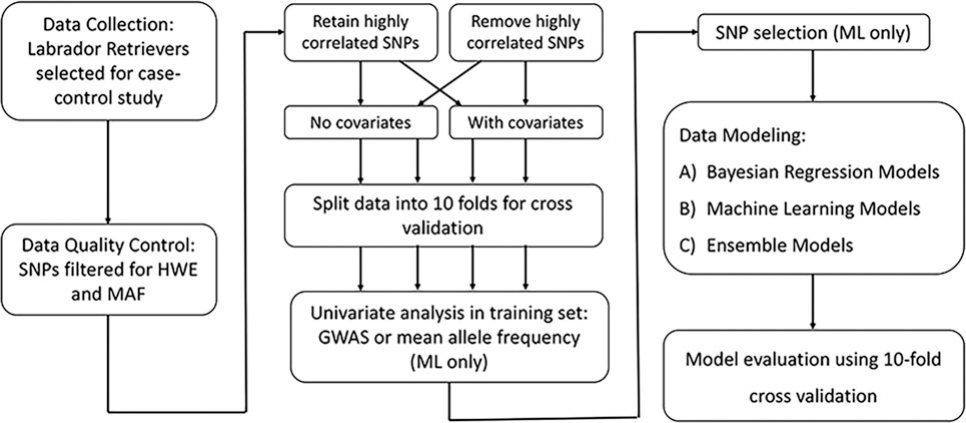
Schematic of data analysis and modeling workflow. HWE: Hardy-Weinberg equilibrium; MAF: minor allele frequency; SNP: single nucleotide polymorphism; GWAS: genome-wide association study; ML: machine learning.

Exploration of the performance of Bayesian and classification-based machine-learning methods for predicting ACL rupture in Labrador Retrievers was evaluated using a 10-fold cross validation framework (Figure 1). In 10-fold cross validation, data are randomly split into 10 partitions, which remained fixed for all methods. In each fold of the validation, one partition is used as the test data set and the other nine partitions are used as the training dataset. The partition scheme used was similar to that in Gianola *et al.* (2011) and González-Camacho *et al.* (2012). This procedure is repeated 10 times so that each fold is predicted once, using the other 9 folds as training data. The advantage of multiple-fold cross validation is that it allows the training dataset to remain large without sacrificing a portion of the dataset for testing, which is very useful especially when the whole dataset is small.

Data were split into folds before implementing feature selection for the models. Care was taken to ensure that feature selection was performed only with consideration to the training set without knowledge of the test set for each fold. The predictions were aggregated from the 10 folds and averaged across the runs. Prediction performance was scored using area under the ROC curve (AUC). This process was repeated 5 times for each model evaluated. Models were compared using the average AUC and standard deviation.

#### Removal of highly correlated SNPs:

Linkage disequilibrium (LD) is extensive in purebred dog populations (Sutter *et al.* 2004). In genomic prediction, SNPs that are in LD with the risk loci serve as surrogates in the model. In some genomic prediction applications, however, the strong LD among SNPs may lead to diminished importance of the true risk loci or tag SNPs in the model, as their effects may end up being partially captured by many SNPs. To mitigate this effect, SNPs with LD r^2^ greater than 0.7 were pruned using PLINK with a window size of 50 SNPs and overlap of 5 SNPs until no pairs remained. LD pruning was performed using the complete dataset before the dataset was split into folds. All models were compared with and without removal of highly correlated SNPs.

#### Covariates:

Covariates used in the study were known risk factors for ACL rupture in dogs: weight, sex, and neuter status (castration and ovariohysterectomy in males and females, respectively) (Witsberger *et al.* 2008). While age data were also available, it was not considered as a covariate because age was part of the criteria used for selection of dogs to participate in the study. When age is considered as part of the model, this variable provides information about case or control status and ultimately biases predictive accuracy. Covariates were incorporated as additional features in each classification method alongside SNPs. Covariates were also evaluated independently as predictors of ACL rupture using 10-fold cross validation using a logistic regression model. The R package ‘stats’ (R Core Team 2013) was used for implementation of the logistic regression function.

### Bayesian analyses

Genomic prediction models were fitted using five Bayesian logistic model specifications: Bayesian ridge regression, Bayesian LASSO regression, Bayes A, Bayes B, and Bayes Cπ (Gianola 2013). For each sample the genotypic predictors were defined as$\;m_{ij}$ with i=1,…,n, and j=1,…,p for genotypic values and the response vector $y=\{y_{i}\}$ defined as two possible values including presence $y_{i}=1$ or absence $y_{i}=0$ of ACL rupture for the i^th^ individual. A probit link function $\text{P}\left(y_{i}=1\text{|}\mu ,\alpha \;\right)=\text{Φ}\left(\eta_{i}\right)$ was used where, Φ is a standard normal cumulative distribution function (CDF) and $\eta_{i}$ is a linear predictor given by:

$$\eta_{i}=1\mu +Xb+\overset{p}{\underset{j=1}{\sum }}m_{ij}\alpha_{j}+e$$

Above, µ is an intercept, X is an incidence matrix of the fixed effects in b (weight, sex, and neutered status), p is the number of markers fitted, $m_{ij}$ is the genotype of the i^th^ individual at the j^th^ SNP marker, and $\alpha_{j}$ is the j^th^ marker effect, and e is a vector of residual effects. Following Albert and Chib (1993) and Lee *et al.* (2003), the probit link function was implemented using a latent normally distributed variable $l_{i}=\eta_{i}+\varepsilon_{i}$ and assumed that

$$y_{i}=\{\begin{array}{c} 1\;\;\;\;\;if\;\;\;\;\eta_{i}>\;\gamma \\ 0\;\;\;\;\;if\;\;\;\;\eta_{i}\leq \;\gamma \end{array}$$

where γ is a threshold parameter; $\varepsilon_{i}$ is an independent normal model residual with mean zero and with variance set equal to 1 as the parameter is not likelihood identified. To perform variable selection, a vector δ of p indicator variables is introduced:

$$\delta_{j}=\{\begin{array}{c} 1\;\;\;\;\;if\;\;\;\;\alpha_{j}\neq 0\;\;\;\;\;\;\;\;\;\;\;variable\;j\;selected\\ 0\;\;\;\;\;if\;\;\;\;\alpha_{j}=0\;\;\;\;variable\;j\;not\;selected \end{array}$$

A standard Bayesian linear model was used for whole genome prediction using binary data, as follows:

$$p\left(\theta_{M}\text{|}y,\;\omega_{M}\right)\propto p\left(y\text{|}\theta_{M}\right)\;p(\theta_{M}|\omega_{M})$$

where $p(\theta_{M}|y,\;\omega_{M})$ is the conditional posterior density of the genomic parameters ($\theta_{M}$); µ was assigned a flat prior density, and the marker effects (α) were assigned independent and identically distributed informative priors, depending on the model; $\omega_{M}$ represents the genomic hyperparameters. The expression $p\left(y\text{|}\theta_{M}\right)=\overset{n}{\underset{1}{\prod }}\left\{{\left[\text{Φ}(\eta_{i})\right]}^{y_{i}}{\left[1-\text{Φ}(\eta_{i})\right]}^{1-y_{i}}\right\}$ is the conditional distribution of the phenotype given the linear predictor, and $p\left(\theta_{M}\text{|}\omega_{M}\right)\propto p\left(\alpha_{j}\text{|}\omega_{M}\right)\;p(\sigma_{e}^{2})$ is the joint prior distribution of model unknowns, given the hyperparameters. The prior density of marker effects, $p\left(\alpha_{j}\text{|}\omega_{M}\right)$, defines the specification of the various Bayesian methods inducing shrinkage and variable selection (Bayes B and Bayes Cπ have a scaled-*t* and a Gaussian prior, respectively), or shrinkage only (Bayes A, BRR, and BL with scaled-*t*, Gaussian and Laplace priors, respectively). For more details, see de los Campos *et al.* (2013). Models were run using the BGLR statistical package (Pérez and de Los Campo 2014) in R (www.Rproject.org) for a total of 52,000 iterations with the first 6,000 iterations discarded. Each Bayesian model employs different prior assumptions for marker effects. A brief description denoting the difference between the models follows.

#### Bayesian ridge regression:

In Bayesian Ridge Regression (BRR), an independent Gaussian prior with common variance is assigned to each regression coefficient. This scenario assumes that all markers have some effect and shrinkage is applied homogenously across the dataset.

#### Bayesian LASSO regression:

Bayesian *Least Absolute Shrinkage and Selection Operator* (LASSO) regression (Park and Casella 2008), uses a double-exponential or Laplace prior distribution for marker effects. This places a higher mass at zero, meaning it induces a strong shrinkage toward zero. This is a logical application in a situation where most of the many thousands of SNP markers available are assumed to have little or no effect on the trait being tested.

#### Bayes A:

Bayes A (Meuwissen *et al.* 2001) uses a scaled-t prior distribution for marker effects. Similar to Bayesian LASSO, this places a higher mass at zero, inducing strong shrinkage toward zero. The scaled-t distribution places slightly less emphasis on shrinkage toward zero, allowing more flexibility for marker effects than Bayesian LASSO (de los Campos *et al.* 2013).

#### Bayes B:

Bayes B assumes that most of the genetic markers have zero effect, so that the distribution can be described as a mixture model where π is the probability that the SNP has no effect and (1-π) is the probability that the SNP contributes to genetic variance (Meuwissen *et al.* 2001). To run Bayes B, we used default prior rules in BGLR to give a weakly informative prior: ${\text{π}}_{0}$= 0.5 and ${\text{P}}_{0}$ = 10 (de los Campos *et al.* 2013). Non-null marker effects are assumed to have a scaled-t prior distribution, as in Bayes A. Therefore, the model is fairly stringent, assuming that relatively few markers have non-null effects.

#### Bayes Cπ:

Bayes Cπ (Habier *et al.* 2011) is a mixture model similar to Bayes B, where a prior distribution is assumed for the proportion of null effect markers and non-null effect markers. In Bayes Cπ, non-null effect markers are assumed to have a Gaussian prior with a common variance. As with Bayes B, we used default prior rules to run Bayes Cπ: ${\text{π}}_{0}$= 0.5 and ${\text{P}}_{0}$ = 10.

### Machine learning analyses

#### SNP selection:

SNPs were selected for inclusion in the training set by one of two filter methods: 1) ranked *P*-values from a linear mixed model GWAS using the R package ‘gaston’ (Perdry and Dandine-Roulland 2015), where smaller *P*-values were considered more likely to be associated with ACL rupture or 2) ranked SNPs based on the mean difference in allele frequency between cases and controls. SNPs with the largest mean difference were considered to be the most likely associated with ACL rupture (Hajiloo *et al.* 2013). The number of genetic variants believed to affect ACL rupture in dogs is unknown, though there are likely hundreds to thousands of non-null effect SNPs (Baker *et al.* 2017; Baker *et al.* 2018). Therefore, prediction performance of each model was assessed at several SNP inclusion thresholds from 5 to 15,000 SNPs. For each SNP inclusion threshold, the ranked SNPs were chosen using only training data after the test fold was removed from the dataset. This procedure was performed separately for each of the five 10-fold cross validation runs.

#### Classification methods:

Four classification methods were considered in this study. A brief description of each method follows:

##### Weighted subspace random forest

In Random Forest (RF), a collection (“forest”) of separate tree-structured classifiers each cast a vote for the classification of an input and the majority vote of the trees is chosen as the correct classification (Breiman 2001). This method has the benefits of being fast and unlikely to over-fit to the dataset. Further, it is easily optimizable and provides variable importance estimates for further feature refinement. One shortcoming of random forest for high-dimensional data are the random selection of features which can fail to consistently select informative features. To address this issue, weighted subspace random forest (wRF) was used in the final validation of the methods. wRF weights each of the SNPs based on correlation of the SNP with the case or control class. It then calculates probability based on weights and uses it for variable selection (Zhao *et al.* 2017). wRF was implemented using the R package ‘wsrf’ (Zhao *et al.* 2017). Models were built using at least 1000 trees and the square root of the total number of features at each tree split.

##### Gradient boosted trees

Similar to RF, gradient boosted trees (GBT) uses an ensemble of tree-based classifiers for phenotype prediction. However, instead of creating decision trees independently of the other trees, trees are created conceptually in serial order, with each new tree attempting to minimize the mean squared error of the previous trees (Natekin and Knoll 2013). Gradient boosting theoretically provides an advantage over random forest at the cost of greater computational complexity and the need to tune hyperparameters. The R package ‘xgboost’ (Chen *et al.* 2015) was used for implementation of gradient boosted trees. Tuning of the hyperparameters was performed using fivefold cross validation grid search techniques. The cross validation function from xgboost was used to determine the number of rounds to run the algorithm. The hyperparameters used were learning rate *eta =* 0.05, minimum loss reduction *gamma =* 0.3, maximum tree depth = 10, subsample ratio of columns when constructing trees = 0.8, subsample ratio of training instances = 0.8 and evaluation metric of binary classification error rate with 1000 rounds of training.

##### Naïve bayes

One of the first machine learning methods used in bioinformatics, Naïve Bayes (NB) is a classification method based on Bayes’ theorem. A training set is used to calculate frequencies of genotypes in case or control individuals, and this information is used to calculate the probability of an unknown individual’s classification. NB is known for being simple and computationally efficient, but it is prone to miscalibration when features are high in number, as is the case with SNP datasets (Acikel *et al.* 2016). Though it has been theoretically outclassed by ensemble machine learning methods, NB is still an excellent baseline for comparing classifiers (Acikel *et al.* 2016). The R package ‘e1071’ (Dimitriadou *et al.* 2017) was used for NB implementation.

##### K-nearest neighbors

K-nearest neighbors (KNN) is the most simplistic classifier, as it does not build a classifier using the training data. Instead, KNN compares the unknown input with classification of the *k*-nearest data points and uses the features of these neighbors to classify the unknown input. If multiple classifications are possible, a majority vote is applied (Acikel *et al.* 2016). However, KNN also struggles when the number of inputs is very large. Because this method does not depend on training and tuning, it serves as another baseline method for comparing other classifiers. The R package ‘caret’ (Kuhn 2008) was used for KNN implementation. Models considered the five closest neighbors for classification decisions.

##### Ensemble learning

Ensemble learning methods were applied to determine whether better predictive performance could be obtained when multiple classifiers are considered in aggregate. Two methods of ensemble learning were used, 1) n-agreement and 2) supervisory learning.

When the four machine learning algorithms described above were used with two methods of feature selection, a total of 8 base-level models were considered. For our n-agreement ensemble approach, we defined an ensemble agreement threshold at each integer **n** between 1 and 8, rendering a positive prediction if and only if at least n of the 8 base models agree on a positive prediction. This n-agreement ensemble was applied on each fold within the cross validation workflow at each integer value of n between 1 and 8. Within each fold, the value of n was saved for the scenario with the maximum AUC. The value of n and the maximum AUC were averaged across the 10 folds and 10-fold cross validation was repeated five times.

In the supervisory machine learning approach, predictions from each of the 8 base-level learners were used as features in 1) logistic regression or 2) random forest models. The cross validation workflow was extended for this method. In this framework wRF, GBT, NB, and KNN models were trained using 10-fold cross validation. Then, the aggregated predictions from these models were randomly re-ordered and re-partitioned into 10 new folds and employed as predictors in an additional 10 fold cross-validation experiment using logistic regression and random forest models. The concept of training a prediction model using predictions of lower-level models as its features is called “stacked” ensembling, and is a well-established procedure (Wolpert 1992). This protocol was also repeated five times for each supervisory model.

### Data availability

Genotype data, phenotype data, and all code that was used for these analyses are available at figshare: https://figshare.com/s/c40ae1baf8cb4333ed57. Genotype and phenotype data are presented in PLINK binary (.bed, .bim, .fam) format. The bed files contain genotyping information in binary format. The bim files contain SNP information. The fam files contain phenotype information for each dog. Both complete and LD-pruned datasets are available. LD-pruned datasets have “0.7” in the filename to indicate that SNPs with LD r^2^ > 0.7 have been removed. Supplementary material describing an alternate method and results for ensemble learning is also available at figshare (File S1). Supplemental material available at figshare: https://doi.org/10.25387/g3.12001344.

## Results

The final dataset included 622 Labrador Retriever dogs (247 cases and 375 controls). Among cases, there were 14 intact females, 25 intact males, 111 ovariohysterectomized females, and 97 castrated males. Among controls, there were 59 intact females, 65 intact males, 130 ovariohysterectomized females, and 121 castrated males. After SNP data quality control, 126,678 SNPs remained. After removing highly correlated SNPs from the dataset, 76,767 SNPs remained.

### Bayesian analyses

**Figure 2:**
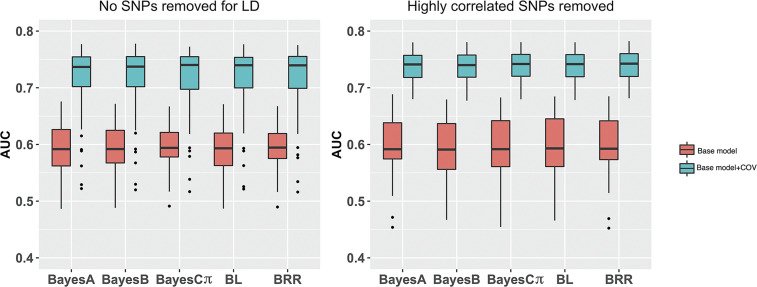
10-fold cross validation of Labrador Retriever SNPs performed with Bayesian genomic prediction models. Averages for model prediction across all folds for 5 repeats per model are reported. Charts show model performance with (base model+COV) and without (base model) inclusion of covariates. The graphs compare model performance with and without removal of highly correlated SNPs prior to analysis.

The prediction accuracy for the Bayesian models described is shown in Figure 2. Model performance was nearly identical across the different types of Bayesian models in each scenario. Including covariates in the model improved prediction accuracy. Removal of highly correlated SNPs did not appear to have an effect on overall prediction accuracy, though it did appear to decrease variability of the estimate when covariates were considered.

### Machine learning analyses

**Figure 3:**
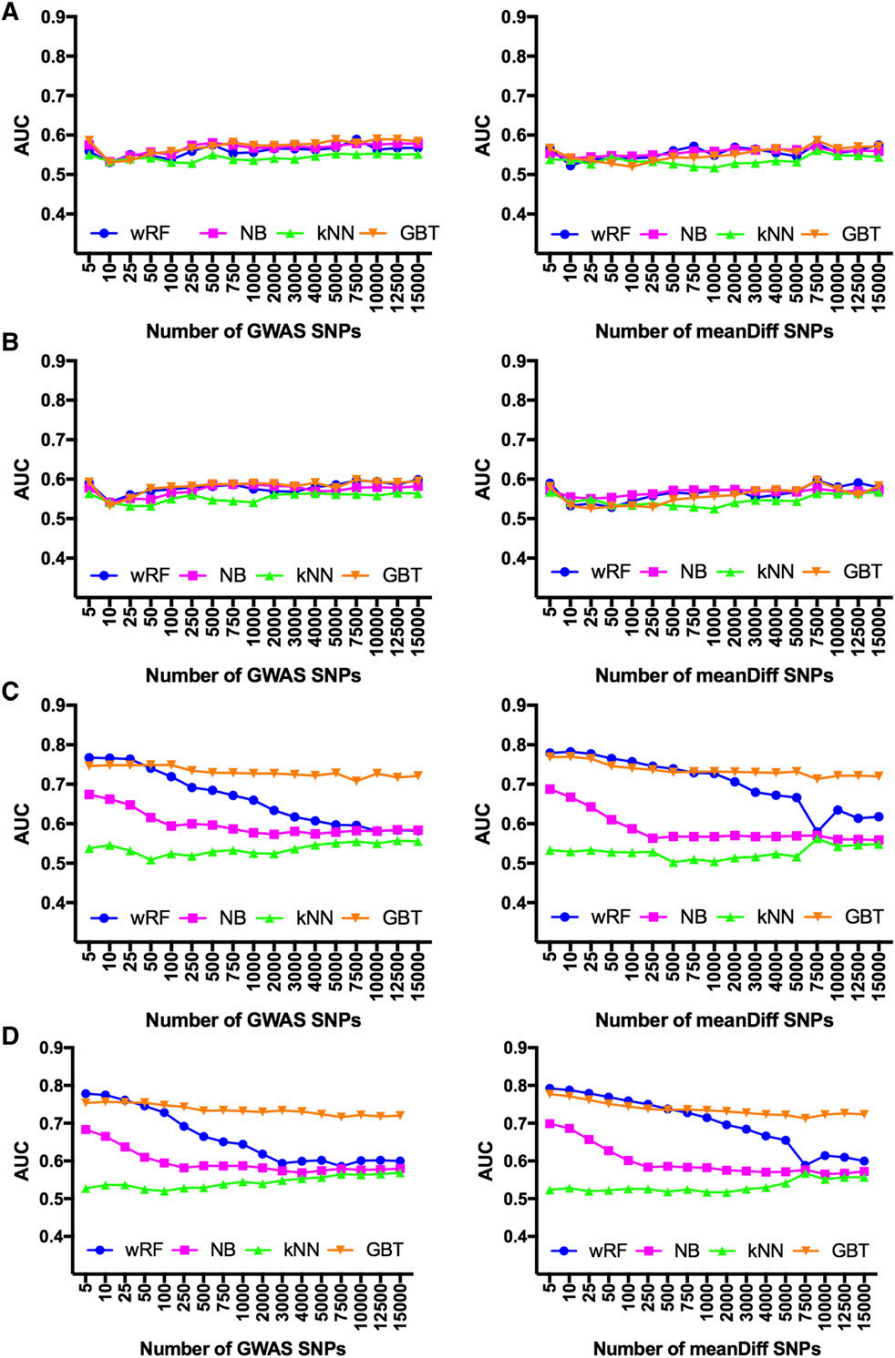
10-fold cross validation of Labrador Retriever SNPs was performed with models trained on feature sets from 5 to 15,000 SNPs. Averages for model prediction across all folds over five runs per model are reported. This analysis used n = 247 cases and n = 375 controls. A. Base model performance without LD pruning or covariates; B. Model performance after LD pruning was performed at r^2^ > 0.7. C. Model performance with covariates (weight, sex, neutering) considered as additional features. D. Model performance with LD pruning and covariates. AUC: area under the ROC curve; wRF: weighted subspace random forest; NB: Naïve Bayes; kNN: *k*-nearest neighbor; GBT: gradient boosted trees.

Results of 10-fold cross validation experiments for machine-learning models are summarized in Table 1. In general, models performed similarly regardless of the model chosen or methods used for feature selection. When LD pruning was not performed and covariates were not considered, the best performing model was GBT with 10,000 SNPs derived from GWAS analysis (AUC = 0.590 (0.049)). Removal of highly correlated SNPs through LD pruning did not have a significant effect on classifier performance, though the same level of performance was achieved with fewer SNPs for some models. Including covariates as predictors accentuated the performance of the classifiers, both with and without LD pruning. When covariates were not considered, model performance improved slightly as more SNPs were added to the model. Once covariates were included, however, model performance tended to decrease with increasing numbers of SNPs (Figure 3). The best performing model overall was wRF with 5 SNPs chosen through mean difference (AUC = 0.792 (0.027)).

**Table 1:** Highest performing machine learning models in 10-fold cross validation for prediction of ACL rupture in Labrador Retriever dogs

Model	Feature Selection	No. SNPs	AUC (SD)
*No SNPs removed for LD*; *Covariates not considered*			
wRF	GWAS	7500	0.584 (0.048)
	meanDiff	7500	0.572 (0.059)
GBT	GWAS	10000	0.590 (0.049)
	meanDiff	7500	0.588 (0.059)
NB	GWAS	7500	0.584 (0.025)
	meanDiff	7500	0.584 (0.055)
KNN	GWAS	10000	0.553 (0.045)
	meanDiff	7500	0.564 (0.039)
*Highly correlated SNPs removed*; *Covariates not considered*			
wRF	GWAS	15000	0.599 (0.050)
	meanDiff	7500	0.598 (0.056)
GBT	GWAS	7500	0.599 (0.039)
	meanDiff	7500	0.597 (0.040)
NB	GWAS	750	0.587 (0.054)
	meanDiff	7500	0.576 (0.036)
KNN	GWAS	12500	0.565 (0.052)
	meanDiff	5	0.567 (0.045)
*No SNPs removed for LD*; *Covariates added to model*			
wRF	GWAS	10	0.782 (0.035)
	meanDiff	5	0.767 (0.034)
GBT	GWAS	10	0.770 (0.050)
	meanDiff	100	0.749 (0.037)
NB	GWAS	5	0.688 (0.033)
	meanDiff	5	0.674 (0.038)
KNN	GWAS	7500	0.562 (0.034)
	meanDiff	12500	0.557 (0.039)
*Highly correlated SNPs removed*; *Covariates added to model*			
wRF	GWAS	5	0.778 (0.025)
	meanDiff	5	0.792 (0.027)
GBT	GWAS	10	0.757 (0.027)
	meanDiff	5	0.777 (0.031)
NB	GWAS	5	0.683 (0.031)
	meanDiff	5	0.699 (0.040)
KNN	GWAS	15000	0.569 (0.038)
	meanDiff	7500	0.567 (0.044)

wRF = weighted random forest; GBT = gradient boosted trees; NB = Naïve Bayes; KNN = K nearest neighbors; AUC = Area under the ROC curve.

### Ensemble learning

Ensemble learning did not result in gains in performance when compared to base learners in 10-fold cross validation (Table 2). In all scenarios, supervisory learning using logistic regression outperformed random forest and n-agreement. Overall, the best performing supervisory model was logistic regression when base models were trained on 100 SNPs (AUC = 0.703 (0.08)).

**Table 2:** Highest performing ensemble models in 10-fold cross validation

Ensemble	N (SD)	No. SNPs	AUC (SD)
*No SNPs removed for LD*; *Covariates not considered*			
nAgreement	5.86 (1.31)	12500	0.598 (0.04)
GLM	N/A	7500	0.611 (0.07)
RF	N/A	5	0.583 (0.09)
*Highly correlated SNPs removed*; *Covariates not considered*			
nAgreement	5.68 (1.17)	4000	0.607 (0.04)
GLM	N/A	4000	0.611 (0.06)
RF	N/A	5	0.579 (0.09)
*No SNPs removed for LD*; *Covariates added to model*			
nAgreement	5.14 (0.76)	5	0.687 (0.07)
GLM	N/A	25	0.695 (0.09)
RF	N/A	5	0.692 (0.09)
*Highly correlated SNPs removed*; *Covariates added to model*			
nAgreement	5.24 (0.96)	10	0.694 (0.07)
GLM	N/A	100	0.703 (0.08)
RF	N/A	5	0.702 (0.09)

N = average number of models that agreed on the prediction; AUC = area under the ROC curve; GLM = supervisory learning with logistic regression; RF = random forest.

### Covariate analysis

10-fold cross validation using a logistic regression model of sex, neuter status, and body weight reached an AUC = 0.734 (0.032).

## Discussion

This work demonstrates that it is feasible to predict ACL rupture using SNP data and relevant covariates from dogs within the Labrador Retriever breed with a sufficient sample size. For all models except KNN, the best predictions were achieved when covariates were considered in the analysis. This is reasonable, as the heritability of ACL rupture in dogs has been estimated between 0.3 and 0.5 (Nielen *et al.* 2001; Wilke *et al.* 2006; Baker *et al.* 2017), which means a substantial proportion of variance for ACL rupture is explained through environmental effects. When the genomic profile is considered alone, the maximum AUC that can be achieved in a classifying algorithm is dependent upon heritability of the trait and disease prevalence. As the disease prevalence of ACL rupture in the Labrador Retriever is 0.0579 (Witsberger *et al.* 2008), the maximum achievable AUC in a model that explains 100% of genetic variance, assuming a heritability of 0.4, is 0.86 (Wray *et al.* 2010). Given our relatively small sample size, the density of our SNP dataset, and prior evidence supporting the hypothesis that ACL rupture is highly polygenic (Baird *et al.* 2014; Baker *et al.* 2017), it is unlikely that we can explain 100% of genetic variance, and therefore, while the AUC we were able to achieve using SNP data alone appears relatively poor, it is reasonable given the heritability and prevalence of ACL rupture in the Labrador Retriever population. Notably, the maximum AUC that can be achieved with a genomic profile that explains one quarter of genetic variance is 0.69, which is closer to the estimates achieved in this exploratory analysis, but indicates that much of the genetic variance of ACL rupture in this population remains unexplained by our genotyping dataset.

We performed genomic prediction using five Bayesian regression models that differed principally in the prior chosen for the effect distribution of the SNPs. In this study, the prediction performance across these five Bayesian models was roughly equivalent. This result was not entirely unexpected; while the prior chosen for a Bayesian model has been reported to influence inference of individual marker effects, predictive performance across models tends to be similar as long as they are tuned appropriately (Gianola 2013). It should also be noted that there is a mismatch between the prior assumptions used by these models and the genetic architecture of ACL rupture. ACL rupture is expected to be highly polygenic (Baker *et al.* 2017; Baker *et al.* 2018) and none of the priors used for the Bayesian models tested in this study model a polygenic architecture, where many SNPs are expected to have some effect, most with a very small effect size. Therefore, it is logical that no Bayesian model stood out in comparison to the others, as no model has the advantage of a prior that matched expected distribution of SNP effects.

Prediction performance of the machine learning models was similar to the Bayesian models, with the best-performing classifiers slightly out-performing Bayesian regression. When covariates were not considered, all models performed similarly. All models except for KNN showed increased performance when covariates were included as features in the model, and in these scenarios, peak prediction performance was achieved with 5-10 SNPs. Of the classifiers, GBT and wRF tended to out-perform the simpler classifiers. Both NB and KNN struggle when the number of inputs is large, so their weakness here is perhaps unsurprising. Overall, the best performing model was GBT, and its performance remained fairly consistent as more SNPs were included as model features.

When covariates were considered independently, the average AUC achieved was only slightly lower than the top-performing classifiers. This indicates that the majority of the accuracy of prediction is relying on the inclusion of covariate risk factors for ACL rupture, though a small number of SNPs may be providing data that are sufficiently informative to improve prediction. It should be noted that dog weight is itself a complex trait that is partly genetically determined, so the covariates included in this study may also be capturing genetic effects at some level. Two of the ACL rupture risk factors that were included in this study are modifiable variables (dog weight and whether a dog was neutered). Ideally, a genomic prediction algorithm would identify high-risk dogs without these variables, so that clinical action could be taken to reduce risk. For example, the link between neutering and ACL rupture may only refer to dogs who are neutered before one year of age, which is common clinical practice (Torres de la Riva *et al.* 2013). Neutering could then be delayed for dogs at high risk of ACL rupture. Age of neutering was not recorded for the present data. A similar approach could apply to counseling owners on the importance of maintaining a healthy adult weight. This is an important consideration for future models, which should try to capture as much genetic variance as possible so the model will rely less on covariates for predictive accuracy.

Extensive within breed LD (Sutter *et al.* 2004; Karlsson and Lindblad-Toh 2008) means that many SNPs that are highly correlated offer the same information to the model. Through the use of LD pruning, highly correlated SNPs are removed from the feature set, thereby allowing for a greater number of unique SNPs to be considered in the model. We found that LD pruning of SNPs had little effect on overall prediction accuracy, but in some scenarios decreased variability of the estimate. It is notable that in comparison to other purebred dogs, LD in the Labrador Retriever is less extensive. The average haplotype block size in the Labrador Retriever is 20kb, while many other breeds have an average haplotype block size of 1Mb or greater (Gray *et al.* 2009). Therefore, while removing highly correlated SNPs in this dataset did not appear to have a large effect on overall performance of prediction models, this step could be critical for some other dog breeds. This, in combination with the observed reduced variability, leads us to recommend that future models for genomic prediction in dogs should include LD filtering as part of data quality control.

Our machine learning approach implemented feature selection based solely on univariate filtering methods. In most cases, there was little to no difference in model performance between feature selection performed by GWAS or mean difference. When genotypic data are considered alone, some cases showed similar model performance with a smaller number of SNPs when mean difference was used for feature selection. By definition, mean difference chooses SNPs where there is a larger difference between cases and controls, and so it is logical that ranking SNPs in this way may be advantageous when choosing SNPs for case-control classification.

In this study, adding an additional decision-making layer through an ensemble learning approach did not lead to an appreciable gain in prediction performance, and often performed worse than some individual base models. Since ensembles often provide a performance improvement when inputs are uncorrelated, we suspect that correlation among base model outputs was high enough to prevent ensembles from having any benefit. We chose a stacked ensemble approach to perform supervisory machine learning models, as we were interested to learn whether a combination of the base level learners we used might improve prediction performance. We considered another common method for ensemble learning, where the data are partitioned and the testing set is used to calibrate the ensemble, evaluating accuracy in the left-out testing set. This method also did not lead to a gain in prediction performance. Methods and results for this approach are presented in supplementary material, see File S1.

There were several limitations to this study. The sample size used for this research limits the predictive capacity of the models tested, especially when applied to Bayesian regression where sample sizes in the thousands are often needed to accurately estimate SNP effects (de los Campos *et al.* 2013). Although model prediction accuracy for ACL rupture may be clinically significant in our population of Labrador Retrievers, increasing sample size and improving feature selection may further improve performance and validate use of classification-based machine learning methods for ACL rupture prediction within the breed.

In conclusion, genomic prediction of ACL rupture risk in the Labrador Retriever breed can be achieved with clinically relevant accuracy. This manuscript comprises the first attempt at such a feat. Future prediction models in dog populations should use a dense training set with a large sample size, implement LD pruning as a part of data quality control, and mean difference in feature selection. A prediction model for ACL rupture in dogs would allow for selective breeding against ACL rupture and also provide the opportunity for a precision medicine approach to clinical management of high-risk dogs. One goal of this research would be to develop generalized models that can accurately predict ACL rupture in all high-risk breeds, such as the Labrador Retriever, Rottweiler, and Newfoundland (Witsberger *et al.* 2008). Genomic prediction across ancestral populations (breeds) is likely to be much more challenging. The ultimate goal of this work is to develop the dog as a spontaneous disease model for human ACL rupture research. This work comprises a part of that goal, as the ability to accurately assess genetic risk for ACL rupture in the dog would also provide opportunities for clinical trials of disease-modifying therapy that would benefit both canine and human health.
